# Adult attitudes to sustainable dentistry in Trinidad and Tobago and their willingness to accept alternatives

**DOI:** 10.1038/s41405-024-00216-5

**Published:** 2024-05-01

**Authors:** Trudee Hoyte, Akini James, Deysha Carr, Abbinah Donatien Andrew Teelucksingh, Peter Mossey

**Affiliations:** 1 School of Dentistry, Faculty of Medical Sciences, The University of The West Indies, St. Augustine, Trinidad; 2 School of Dentistry, University of Dundee, Park Place, Dundee, DD14HN Scotland

**Keywords:** Health care, Dentistry

## Abstract

**Introduction:**

Attitudes towards and willingness to accept alternatives for sustainable dentistry in Trinidad and Tobago have never been assessed. Market research aids in the understanding of the behaviours of people. Since change can be enacted by public pressure, it is worth engaging the public through research to understand their attitudes and which changes they are willing to accept.

**Method:**

A self-administered questionnaire was distributed to private and public dental clinics. The questionnaire assessed attitude towards and willingness to accept alternatives which decrease the effect of dental treatment on the environment.

**Results:**

The study consisted of 1267 participants. Participants were mostly female, older, employed and mainly of African descent. Participants reported a very positive attitude towards sustainable dentistry (Mean = 3.89, SD = 0.8). and were moderately willing to accept alternatives such as a longer appointment time (Mean 3.47, SD = 0.73) and pay more for their dental treatments (Mean=3.00, SD = 0.87). There was a strong positive correlation with attitudes to sustainable dentistry and participants willingness to accept alternatives such as a longer appointment time (r = 0.658, *p* < 0.05).

**Conclusion:**

The adult population had an overall positive attitude towards sustainable dentistry and was willing to accept alternatives so that their dental treatment would have less impact on the environment.

## Introduction

Sustainable dentistry can be defined as dentistry that seeks to decrease the harmful impact of dental treatment on the environment whilst still adhering to the regulations and standards of dentistry in individual countries. Dentistry has a significant and consistent impact on the environment. This is due to dental practices using large amounts of water, electricity, and generating mercury, plastic and other hazardous waste. The biomaterials used also have a substantial environmental impact [1]. The environment and health are closely linked, and dentists and other healthcare professionals should, therefore, adhere to the Hippocratic oath of ‘first do no harm’. Healthcare therefore has an intrinsic responsibility to prevent adverse environmental effects [2]. Global surface temperatures are expected to increase by 1.5 °C by 2050 due to human activities [3]. Record levels of global greenhouse gases have been reached. Reduce, Re-think, Recycle and Reuse are the key ideologies for going green [4]. Dental practices must aim to decrease waste and electricity use, conserve water and use green products. Notwithstanding increased interest in sustainability, there is an apparent uncertainty surrounding the essence of the problem, contributing factors and methods to address them [5].

Moreover, in 2014, patient and staff travel contributed 60% of the national dental carbon footprint in the United Kingdom [6]. In the UK, the NHS is legally required to reduce their greenhouse emissions to net zero by 2050 [7]. Baird et al. [8] conducted an exploratory study similar to this one in the U.K., a society that is advanced in implementing sustainability measures [8]. The healthcare system in Trinidad and Tobago is in the beginning stages, compared to the U.K., of implementing sustainability measures. This is the first study in dentistry on sustainability in Trinidad and Tobago. It is therefore important to compare results from this study to other societies like the U.K. Since instilling an environmentalist mindset is extremely important in these current times and baseline comparative studies will aid in charting the course. Dental practices will need to deliver their services in a socially responsible manner [5, 8].

Multi-stakeholder engagement is necessary to tackle this problem [8–10]. These stakeholders include dentists, patients, dental manufacturers, supply companies and policy makers. Engagement of these stakeholders will prompt opinions of participants at a particular point in time and cause them to deliberate over sustainable dentistry [10].This is also necessary between organizations and individuals to decrease inadvertent outcomes and consequences and drive change. Specifically, we would like changes made for sustainable dentistry to be accepted/ implemented by the stakeholders. Therefore engagement will allow accomplishment of long-term and meaningful improvements in sustainable dental services [8].

Market research aids in understanding the behaviours and views of people [11]. It is well known that change can be enacted by public pressure [12, 13]. It is, therefore, worth engaging the public to understand which changes would be accepted [8]. Therefore, this study explored attitudes towards sustainable dentistry and participants willingness to accept alternatives so that their dental treatment does not have a negative impact on the environment among adults in Trinidad and Tobago. Participants’ attitudes and willingness to accept alternatives would be evaluated through their responses to several questions assessing their willingness to accept other options like a longer appointment time, their willingness to pay more for dental treatment, willingness to accept alternatives to replacing restorations or different options in aesthetic dentistry, and willingness to accept options that may not be ideal for their dental health for sustainability or would that be a step too far.

Some examples of how sustainability can be introduced in dental practices can be in the areas of travel, efficient use of time, reduction of waste, and energy efficiency. Travel from staff and patients accounts for 65% of the carbon dioxide emissions in dentistry. To aid in reducing this dental practices should pursue and encourage more preventative care, combine family appointments, and clinicians can use remote consultations [14].

Having longer appointments can be the alternative relating to time and convenience. This would also decrease the amount of waste by decreasing the number of single-use plastics per patient [8]. Longer appointments can also facilitate multiple procedures(including quadrant dentistry) but would require patients pay more per visit [8]. Also, a practice transitioning to be more sustainable would incorporate the use of digital technology both in the clinic and administration areas of the practice. This is a financial investment which would increase operating costs but would make the practice more efficient and decrease waste due to paper(scratch pads, paper prints, internal notes). It also saves time in updating patient records and makes the practice efficient since there would be faster access to information and it reduces the need for physical storage space [15]. This reduces the environmental impact and enhances patient care. Another important component of dental emissions is energy. Dental practices can make use of low-energy lightning (LED lights), low energy equipment, reduce energy by switching off lights and computers not in use and use green energy(e.g. solar energy to power offices). These energy efficient measures will keep costs down [16].

Defective restorations can be repaired as opposed to being replaced but this may affect the durability of the restoration [17]. Participants willingness to have a restoration that is not tooth coloured will demonstrate their willingness to accept options in the area of aesthetics [8].

This span of possible substitutions allows comparisons of participants willingness to accept alternatives.

## Methods

### Design

A self-administered questionnaire was used to measure attitudes towards sustainable dentistry and participants willingness to accept alternatives to reduce environmental impact of their dental treatment. This cross-sectional study was distributed to a convenience sample of patients attending dental clinics across the twin island republic. Dentists across the islands in private and public clinics were contacted via email, and their participation was requested. Dentists who agreed to participate had questionnaires delivered to their practice. Participants had to be 18 years and older and residents to be eligible to participate.

### IRB approval and informed consent

Approval was obtained from The University of The West Indies ethics committee (Ref: CREC-SA.1835/11/2022) and the regional health authority ethics committee. The front page of the questionnaire defined sustainable dentistry. It advised participants that participation was anonymous and voluntary and that by continuing to the following page, respondents were confirming that they were over 18 and consented to participate in the study.

### Development of survey instrument

The survey was based on the survey used by Baird et al. in the U.K. [8]. It was edited to be relevant to the Trinidad and Tobago population. The actual survey used can be found in the Supplementary Material.

The questionnaire first consisted of demographic questions followed by questions on participants attitude to sustainable dentistry and willingness to accept alternatives. The demographic data consisted of their gender, age, level of education, ethnicity, and employment status. Following this, participants were asked to answer questions about their attitudes and their willingness to accept alternatives that will have a minimal impact on the environment.

### Incentives and timeframe for data collection

There were no incentives offered to participate. Data was collected from February to May 2023. There were at most five items per page. Participants responded to a 5-point Likert scale questions, where 1 represented “strongly agree”, indicating the most positive attitude or the most willingness to accept alternatives for sustainable dentistry, and 5 represented “strongly disagree”, indicating least positive attitude or least willingness to accept alternatives. Responses for the Likert scale were recoded using reverse coding on questions.

### Reliability analysis

Cronbach alpha statistics were calculated for the reliability of the questions/variables and what they measured. Attitudes towards sustainable dentistry(measured-The degree to which participants had a positive attitude towards sustainable dentistry) 0.90. Willingness to accept options such as a longer appointment for sustainable dentistry(measured -The degree to which participants would accept longer appointments to decrease the environmental impact of their dental treatment) 0.844. Willingness to pay more for sustainable dentistry (measured-The degree to which participants would pay more to decrease the environmental impact of their dental treatment) 0.79. Willingness to accept options such as repairing restorations which may affect the durability of dental treatment for more sustainable dentistry(measured -The degree to which participants would accept alternatives to restoration replacement that may affect the durability of dental work to decrease the impact on the environment) 0.48. This item was therefore not as effective and was therefore excluded from further analysis.

Willingness to accept options for aesthetics for more sustainable dentistry (measured -The degree to which participants would accept alternatives for aesthetic dentistry to decrease the environmental impact) 0.79. Willingness to make sacrifices for their dental health for sustainable dentistry (measure—The degree to which participants would sacrifice their dental health to decrease the environmental impact) 0.67.

### Data analysis and statistical procedures

Specific variables were assumed to be associated with each of the predetermined factors: Attitude towards sustainable dentistry, Time and Convenience, Money, Aesthetics, and Health. To confirm if these variables are indeed part of the same underlying group, a factor analysis was conducted. Suspected variables were included in the analysis, and this was repeated for each group. Therefore, all variables suspected to be part of the same underlying group, for example, all the suspected variables related to attitude, were selected for the analysis. The factor loadings were examined to confirm whether the observed variables indeed group together as expected based on theoretical or conceptual considerations. High factor loadings identify which variables are strongly associated with each group. If variables do not load heavily on any factor or load on unexpected factors, the grouping would have been reconsidered.

Subsequently, five quantitative variables were created by calculating the average scores for each participant based on the identified groupings from the factor analysis.

Average scores from the responses for each item in each group were calculated to get one mean value for each group.

A visualization of the data was conducted to observe its distribution before pursuing the relevant statistical test. Normality and other assumptions were checked. Pairwise deletion was employed to handle missing data. Descriptive analysis, Pearson correlation analysis, Independent sample t-test and Analysis of Variance (ANOVA) test followed by the Bonferroni multiple comparison test were conducted. Statistical analyses were conducted using IBM SPSS Statistics 29.0.0.0 and RStudio programming software [18, 19].

## Results

From the demographic data, the sample had a good age spectrum and consisted of mainly females. The participants appeared to be relatively affluent, non-deprived, and highly educated. Most of the participants were Afro-Trinidadian and the participants had a high unemployment rate. Table 1 shows the demographic characteristics of the 1267 participants. The participants were predominantly female (65.6%). The majority were aged between 26–40 years (37.2%). Nearly half were of African descent (41.3%) and the lowest education level was primary (3%), while the majority of participants had an undergraduate degree (29.8%). The majority of participants were employed (72.6%) and attended private dental clinics (93.5%).

**Table 1:** Frequency and distribution of demographics factors.

Category	Missing (%)	Frequency (%)
Sex		
Male	8 (0.6)	429 (33.9)
Female		830 (65.5)
Age Category		
18–25	1 (0.1)	185 (14.6)
26–40		471 (37.2)
41–60		388 (30.6)
>60		222 (17.5)
Ethnicity		
Indo-Trinbagonian	5 (0.4)	431 (34.0)
Afro-Trinbagonian		523 (41.3)
Other		308 (24.3)
Education		
Primary School	1 (0.1)	39 (3.1)
Secondary School		327 (25.8)
Diploma		263 (20.8)
Bachelor’s Degree		377 (29.8)
Postgraduate Degree		260 (20.5)
Employment		
Employed	10 (0.8)	920 (72.6)
Unemployed		337 (26.6)
Clinic		
Private	0 (0.0)	1185 (93.5)
Public		82 (6.5)

The factor analysis confirmed groupings of variables: Attitude, Time and Convenience, Money and Aesthetics. The factor analysis helped confirm that the observed variables do indeed group together as expected based on theoretical or conceptual considerations. All variables weighted heavily on the same factor, indicating that they are part of the same underlying group. This occurred in each grouping.

Descriptive statistics for each study variable are shown in Table 2. Participants reported a very positive attitude towards sustainable dentistry (Mean = 3.89, SD = 0.8) and were moderately willing to accept alternatives such as a longer appointment time(Mean 3.47, SD = 0.73) and pay more for their dental treatments (Mean=3.00, SD = 0.87). It was less clear if they were willing to accept alternatives with the aesthetics of their treatment(Mean= 2.55, SD = 0.83) or their dental health (Mean = 2.16, SD = 0.95).

**Table 2:** Study variables descriptive statistics.

	*N*	Mean	Std. Deviation	95% CI	Min	Max
Attitude towards sustainable dentistry	1265	3.89	0.80	3.84, 3.93	1	5
Willingness to have a longer appointment : Time and Convenience	1265	3.47	0.73	3.43, 3.50	1	5
Willingness to accept alternatives for sustainable dentistry: Pay more	1265	3.00	0.87	2.96, 3.05	1	5
Willingness to accept alternatives for sustainable dentistry: Aesthetics	1265	2.55	0.83	2.50, 2.60	1	5
Willingness to accept alternatives for sustainable dentistry: Dental Health	1265	2.16	0.95	2.11, 2.21	1	5

Correlation between study variables are shown in Table 3. The correlations were all significant except for the correlation between attitudes to sustainable dentistry and willingness to accept alternatives with the appearance of their teeth (r = –0.042, *p* > 0.001). There was a strong positive correlation with attitudes to sustainable dentistry and participants willingness to accept the option of a longer appointment time (r = 0.658, *p* < 0.05) and moderately positive correlation with their willingness to pay more (r = 0.358, *p* < 0.001). There was a significantly negative correlation between attitude to sustainable dentistry and dental health (r = –0.229, *p* < 0.001).There exists a significantly moderate correlation between participants willingness to accept alternatives for sustainable dentistry regarding a longer appointment time and paying more for their dental treatments (r = 0.532, *p* < 0.001). There were no significant correlations with willingness to accept alternatives such as a longer appointment time and their dental health.

**Table 3:** Correlations matrix between study variables.

	1	2	3	4	5	6
1. Attitude towards sustainable dentistry	1					
2. Willingness to accept a longer appointment time	0.658^a^	1				
3. Willingness to pay more for sustainable dentistry: Money	0.358^a^	0.532^a^	1			
4. Willingness to accept alternatives for sustainable dentistry: Aesthetics	−0.042	0.218^a^	0.345^a^	0.500^a^	1	
5. Willingness to accept alternatives for sustainable dentistry: Dental Health	−0.229^a^	−0.027	0.169^a^	0.224^a^	0.504^a^	1

^a^Correlation is significant at the 0.05 level (2-tailed).

Independent sample t-tests and ANOVA tests were conducted to observe statistically significant differences in the mean scores for the variables attitude towards sustainable dentistry and willingness to accept alternative such as a longer appointment, paying more for dental treatments, alternatives to aesthetic dentistry, and their dental health by demographic factors. For the t-test, the appropriate *p* values were used based on Levene’s test for equal variances. Table S1 (in Supplementary Material)shows the t-test and ANOVA results. Amongst gender the table shows that there exist significant differences in the mean scores for attitude to sustainable dentistry (*p* < 0.001), willingness to accept alternatives such as a longer appointment time (*p* = 0.01), aesthetics (*p* = 0.016), and dental health (*p* < 0.001). Descriptive statistics showed that females had a more positive attitude towards sustainable dentistry and were more willing to accept a longer appointment time. Males however were more willing to accept alternatives to dental aesthetics and their dental health.

All study variables showed significant differences amongst age categories (*p* < 0.05) except for willingness to accept alternatives for dental health for sustainable dentistry (*p* = 0.197). Only aesthetics showed no significant differences amongst education (*p* = 0.087), while all other variables showed significant differences (*p* < 0.05). Significant differences only exist for attitude to sustainable dentistry (*p* = 0.01), aesthetics (*p* = 0.02) and dental health amongst ethnic groups (*p* < 0.001). For all variables, there exist no significant differences amongst employment status. Regarding clinics, there only exist significant differences in money (*p* < 0.001) and health (*p* < 0.01). All other variables showed no significant differences.

## Discussion

The aim of this study was to investigate the attitudes of adults and their willingness to accept alternatives for sustainable dentistry in Trinidad and Tobago.

Stakeholder engagement was critical in this research since the literature has shown that there can be widespread awareness about sustainability, but without stakeholder engagement, this does not translate to a behavioural response [10]. Noteworthy is that adults in the USA and UK have reported a patchy knowledge about climate change, and this did not correspond to an awareness of solutions [10, 20]. Despite this, few studies have addressed individuals’ willingness to accept alternatives to be more sustainable.

The study findings were consistent with other studies [8] where participants reported an overall positive attitude towards more sustainable dentistry. They were also willing to accept alternatives and have longer dental appointments and pay more for their dental treatments. This could be due to the participants being the more affluent members of the society since most attended private fee paying clinics. They therefore like the Baird et al. and Lorenzoni et al. studies showed less willingness to accept alternatives in the areas of aesthetics and dental health[8, 10]. In practice participants health would never have been compromised and no researcher would have expected participants to agree to this. This is consistent with Lorenzoni et al. findings whereas environmental sustainability was important, participants considered their dental health more important [10]. In contrast to the Baird et al. study participants in this study were not willing to make sacrifices with the durability of their dental treatments [8].

In our study, there was also a strong positive correlation between participants' willingness to accept alternatives such as a longer appointment time and their attitude to sustainable dentistry. There was also moderate correlation between participants' willingness to accept alternatives such as a longer appointment time and to pay more for their dental treatments. These can be explained by the psychological behavioural model “The theory of planned behaviour” which stipulates that there are factors like social attitude which play a significant role in explaining and predicting human behaviour [21]. The literature states that encouraging positive attitudes is the most excellent forecaster of subsequent behaviour [22].

To help to understand participants' attitude and their willingness to accept alternatives factors such as age, gender, ethnicity, education level, employment status and type of clinic attended were explored.

Looking at the age categories of participants, participants 41–60 and older than 60 years had a greater positive attitude to sustainable dentistry. These findings were consistent with other studies [23]. Also, in these age categories participants were more willing to accept alternatives such as a longer appointment time and pay more for dental treatments but not alternatives to dental aesthetics and dental health. To reduce the impact of their dental care on the environment, the older participants were also willing to pay more for dental care. Conversely, other studies have stated that the younger generation(Millennials and Generation Z) have been reported to be more progressive towards sustainability than the older generation [24].

In this study there was a difference between genders in relation to attitude towards sustainable dentistry. Females showed a more positive attitude to sustainable dentistry and were more willing to accept longer appointment times. Females are heading environmental movements in the political arena around the world. Women display more pro-environmental behaviours than men. This has also been reported by Echavarren [18] et al. and Volgenant et al. [19]. It can be explained by the ecofeminism concept which refers to feminist and women’s views on the environment where women who are poorly resourced, exploited and dominated and nature are all connected. Other authors have reported that males can be made to employ sustainable consumption [25]. Notably in this study males were more willing to accept alternatives to aesthetics and their dental health compared to females.

Pertaining to ethnicity, there was a significant difference between the Indo-Trinidadian and Afro-Trinidadian participants, showing statistical differences in attitudes to sustainable dentistry and willingness to accept alternatives for dental aesthetics and oral health. With Indo-Trinidadians being more likely to do so. This is probably due to differences in income, education and different gender ratios within the sample of participants.

Trinidad and Tobago has large reserves of oil and natural gas, which makes it one of the wealthiest countries in the Caribbean. This twin island republic has free tuition from Kindergarten to University and has a high literacy rate; therefore, these two phenomena will account for the findings showing participants in the sample being highly educated and non-deprived.

The sample also had an unusually high proportion of unemployed participants, a bias that is not reflective of the population. The actual unemployment rate for the country is 4.9%. The sample was a convenience sample and one disadvantage of this type of sampling is that the sample can lack generalizability and is not fully representative of the population that may account for the bias seen in this study towards participants being unemployed.

The study also had a significant bias towards private dental clinics. Private care dentists were the main respondents who agreed to participate in the survey. With private and public clinics, we found that participants who attended private clinics were less willing to pay more for dental procedures. The possible explanation is that patients in private clinics were already paying for their dental care and would not be willing, to pay more. Because of the higher cost of going green, this would mean in our environment, publicly funded clinics would have to possibly ask their clients to contribute financially to the higher cost of treatment and these patients were willing to make such a contribution.

A future strategy to improve attitude to sustainable dentistry and willingness of citizens to accept alternatives is education campaigns to raise awareness and change attitudes [26].This research would, therefore, highlight which particular demographic the educational campaigns should target in this society.

## Limitations

This is the first study in this country to assess attitudes towards sustainable dentistry and willingness to accept alternatives to be more environmentally friendly with dental treatments.

The constitution of this sample consisted of two thirds female and mainly Afro-Trinidadian participants. This is not reflective of this population, where percentage by ethnic group for both Afro and Indo-Trinidadians is around 34%, and all other groups comprise around 30% of the population [27–29]. Therefore, this may limit the generalizability of the results since this gender and ethnic composition would affect the results of the study. The sample consisted of what would be considered a highly educated population. This would influence results when comparing the results obtained from other countries that do not have free education from kindergarten to university.

Twenty-five per cent of the sample was unemployed. This difference is important because sacrifices in terms of paying more for dental treatment will be affected by participants’ socioeconomic status and would have affected responses on the survey [8]. There was also a significant bias from clinic types since most respondents were from a private clinic setting. This would therefore mean they were in a financially good position to pay more for care and this would have influenced responses.

## Conclusion

This study can be seen as a pilot study to assess the awareness and attitudes to sustainable dentistry in Trinidad and Tobago. Generally, a positive attitude and a willingness to accept alternatives to reduce the impact of dental care on the environment were demonstrated. These findings demonstrate the kind of alternatives this population may accept in order to have their dental treatments have less impact on the environment.

### Supplementary information

Supplementary Information

## Data Availability

The databases used and/or analyzed during the current study are available from the corresponding author.
