# Supplementary material for: Adult attitudes to sustainable dentistry in Trinidad and Tobago and their willingness to accept alternatives
Source: BDJ Open. 2024 May 1;10:33. doi: 10.1038/s41405-024-00216-5 (PMC11063214; doi:10.1038/s41405-024-00216-5)
Supplement: Supplementary file 1 — Supplementary Information [file 41405_2024_216_MOESM1_ESM.pdf]

Table S1 Independent t-test and ANOVA

	Test Statistic	p-value	Cohen's d/Partial Eta Squared | Effect Size
**Gender**			
Attitude towards sustainable dentistry	t(1257) = 4.87	< 0.001	0.290
Willingness to accept alternatives for sustainable dentistry: Time and Convenience	t(775.6) =2.56	0.011	0.159
Willingness to accept alternatives make for sustainable dentistry: Money	t(1257) = 0.68	0.500	0.040
Willingness to accept alternatives for sustainable dentistry: Aesthetics	t(1257) = -2.300	0.016	-0.143
Willingness to accept alternatives for sustainable dentistry: Health	t(848.7) = -0.347	<0.001	-0.208
**Age**			
Attitude towards sustainable dentistry	F(3,1260) = 22.67	<0.001	0.051
Willingness to accept alternatives for sustainable dentistry: Time and Convenience	F(3,1260) = 20.74	<0.001	0.047
Willingness to accept alternatives for sustainable dentistry: Money	F(3,1260) = 14.52	< 0.001	0.033
Willingness to accept alternatives for sustainable dentistry: Aesthetics	F(3,1260) = 2.87	0.035	0.007
Willingness to accept alternatives for sustainable dentistry: Health	F(3,1260) = 1.56	0.197	0.004
**Education**			
Attitude towards sustainable dentistry	F(5,1258) = 8.02	<0.001	0.031
Willingness to accept alternatives for sustainable dentistry: Time and Convenience	F(5,1258) = 5.80	<0.001	0.023
Willingness to accept alternatives for sustainable dentistry: Money	F(5,1258) = 6.94	<0.001	0.027
Willingness to accept alternatives for sustainable dentistry: Aesthetics	F(5,1258) = 1.93	0.087	0.008
Willingness to accept alternatives for sustainable dentistry: Health	F(5,1258) = 2.39	0.036	0.009
**Ethnicity**			
Attitude towards sustainable dentistry	F(2,1257) = 4.78	0.009	0.008
Willingness to accept alternatives for sustainable dentistry: Time and Convenience	F(2,1257) = 0.15	0.864	0.000
Willingness to make trade-offs for sustainable dentistry: Money	F(2,1257) = 0.76	0.466	0.001
Willingness to accept alternatives for sustainable dentistry: Aesthetics	F(2,1257) = 3.82	0.022	0.006
Willingness to accept alternatives for sustainable dentistry: Health	F(2,1257) = 8.56	<0.001	0.013
**Employment**			

Attitude towards sustainable dentistry	t(628.9)= 0.40	0.696	0.025
Willingness to accept alternatives for sustainable dentistry: Time and Convenience	t(1254) = 1.49	0.137	0.095
Willingness to accept alternatives for sustainable dentistry: Money	t(1255) = -0.95	0.344	-0.060
Willingness to accept alternatives for sustainable dentistry: Aesthetics	t(689.46) = 1.34	0.181	0.080
Willingness to make trade-offs for sustainable dentistry: Health	t(1254) = -0.39	0.698	-0.025
**Clinic**			
Attitude towards sustainable dentistry	t (1265) = 0.23	0.817	0.026
Willingness to accept alternatives for sustainable dentistry: Time and Convenience	t (1264) = 1.14	0.253	0.131
Willingness to accept alternatives for sustainable dentistry: Money	t (1265) = 3.58	<0.001	0.408
Willingness to accept alternatives for sustainable dentistry: Aesthetics	t (1265) = 3.11	0.002	0.355
Willingness to accept alternatives for sustainable dentistry: Health	t (1264) = 1.91	0.056	0.218

Table S2 Descriptive Statistics for gender

Descriptive Statistics						
Gender		N	Minimum	Maximum	Mean	Std. Deviation
1 Male	Attitude	429	1.00	5.00	3.7414	.81111
	Time	429	1.00	5.00	3.3958	.78758
	Money	429	1.00	5.00	2.9852	.88194
	Durability	429	1.00	5.00	3.0676	.86473
	Aesthetic	429	1.00	5.00	**2.6206**	.81083
	Health	428	1.00	5.00	**2.2839**	.95417
	Valid N (listwise)	428				
2 Female	Attitude	830	1.00	5.00	**3.9707**	.78075
	Time	829	1.00	5.00	**3.5110**	.69332
	Money	830	1.00	5.00	3.0201	.86084
	Durability	830	1.00	5.00	3.0566	.88775
	Aesthetic	830	1.00	5.00	2.5031	.83071
	Health	830	1.00	5.00	2.0880	.93651
	Valid N (listwise)	829				

Questionnaire

This short questionnaire is intended for adults in Trinidad and Tobago to determine their attitudes towards sustainable dentistry as part of the Research Project course of the Faculty of Medicine at UWI St. Augustine. Sustainable dentistry is the practice of being more sustainable, less wasteful, and less harmful towards the environment while promoting public oral health. By practicing sustainable dentistry, we would be able to preserve the environment to a greater extent and lessen the amount of harmful emissions produced.

The information collected from this questionnaire is completely anonymous, confidential and will be used for research purposes only. The study involves completing the attached survey which will take approximately 5 minutes.

By continuing to the next page, you indicate that you are over the age of 18 and consent to participate in the study.

Please answer all the following questions.

How old are you?

☐	☐	☐	☐
18-25	26-40	41-60	>60

What is your sex?

☐ Male ☐ Female

What is your ethnicity?

- ☐ Indian
- ☐ African
- ☐ European
- ☐ Chinese
- ☐ Amerindian
- ☐ Middle Eastern (Lebanese, Syrian, Arab, etc)
- ☐ Venezuelan
- ☐ Unknown or undeclared
- ☐ Other, please specify: ………………………………….

What is the highest level of education you have attained?

- ☐ Primary school
- ☐ Secondary school
- ☐ Diploma
- ☐ Bachelor's Degree
- ☐ Master's Degree
- ☐ Doctorate Degree

What is your current employment status?

☐	☐
Employed	Unemployed

Please select the option that best describes how you feel about the following statements.

Attitudes towards sustainable dentistry (8 items):

1. I do not care about the environmental impact of dental services.

☐	☐	☐	☐	☐
Strongly Agree	Agree	Neutral	Disagree	Strongly Disagree

2. I do not care if my dental treatments harm the environment.

☐	☐	☐	☐	☐
Strongly Agree	Agree	Neutral	Disagree	Strongly Disagree

3. It is important to me that my dental treatments do not harm the environment.

☐	☐	☐	☐	☐
Strongly Agree	Agree	Neutral	Disagree	Strongly Disagree

4. It does not bother me whether or not my dental practice is environmentally friendly.

☐	☐	☐	☐	☐
Strongly Agree	Agree	Neutral	Disagree	Strongly Disagree

5. It is important to me that my dental practice tries to reduce the impact on the environment.

☐	☐	☐	☐	☐
Strongly Agree	Agree	Neutral	Disagree	Strongly Disagree

6. The environmental impact of my dental work is not important to me.

☐	☐	☐	☐	☐
Strongly Agree	Agree	Neutral	Disagree	Strongly Disagree

7. It is worthwhile trying to find a dental practice that is more environmentally friendly.

☐	☐	☐	☐	☐
Strongly Agree	Agree	Neutral	Disagree	Strongly Disagree

8. It is good to look after my teeth to reduce the impact of my dental work on the environment.

☐	☐	☐	☐	☐
Strongly Agree	Agree	Neutral	Disagree	Strongly Disagree

Willingness to make compromises for sustainable dentistry.

Time and convenience (7 items):

1. I would be willing to wait longer for an appointment if my dental treatments were better for the environment.

☐	☐	☐	☐	☐
Strongly Agree	Agree	Neutral	Disagree	Strongly Disagree

2. I would be willing to change my dental practice to one that was more environmentally friendly.

☐	☐	☐	☐	☐
Strongly Agree	Agree	Neutral	Disagree	Strongly Disagree

3. I would be willing to visit the dentist with family if it was better for the environment.

☐	☐	☐	☐	☐
Strongly Agree	Agree	Neutral	Disagree	Strongly Disagree

4. I would be willing to visit a more environmentally friendly dentist even if it was more inconvenient to me.

☐	☐	☐	☐	☐
Strongly Agree	Agree	Neutral	Disagree	Strongly Disagree

5. I would be willing to have a longer appointment if my dental treatments were better for the environment.

☐	☐	☐	☐	☐
Strongly Agree	Agree	Neutral	Disagree	Strongly Disagree

6. I would be willing to visit the dentist less frequently if it was better for the environment.

☐	☐	☐	☐	☐
Strongly Agree	Agree	Neutral	Disagree	Strongly Disagree

7. I would not be willing to go out of my way to reduce the impact of my dental treatments on the environment.

☐	☐	☐	☐	☐
Strongly Agree	Agree	Neutral	Disagree	Strongly Disagree

Money (3 items):

1. I would be willing to pay extra to reduce the impact of my dental treatments on the environment.

☐	☐	☐	☐	☐
Strongly Agree	Agree	Neutral	Disagree	Strongly Disagree

2. I would be willing to pay more if my dental treatments were better for the environment.

☐	☐	☐	☐	☐
Strongly Agree	Agree	Neutral	Disagree	Strongly Disagree

3. I would not be willing to pay more for environmentally friendly dental treatments.

☐	☐	☐	☐	☐
Strongly Agree	Agree	Neutral	Disagree	Strongly Disagree

Durability (3 items):

1. I would be willing to have a filling that didn't last as long if it was better for the environment.

☐	☐	☐	☐	☐
Strongly Agree	Agree	Neutral	Disagree	Strongly Disagree

2. I would not be willing to have a less durable filling even if it was better for the environment.

☐	☐	☐	☐	☐
Strongly Agree	Agree	Neutral	Disagree	Strongly Disagree

3. I would be willing to have a filling repaired, rather than replaced, if it was better for the environment.

☐ Strongly Agree	☐ Agree	☐ Neutral	☐ Disagree	☐ Strongly Disagree

Aesthetics (5 items):

1. I would be willing to compromise the appearance of my teeth if it was better for the environment.

☐ Strongly Agree	☐ Agree	☐ Neutral	☐ Disagree	☐ Strongly Disagree

2. I would be willing to have a less than perfect smile if it was better for the environment.

☐ Strongly Agree	☐ Agree	☐ Neutral	☐ Disagree	☐ Strongly Disagree

3. I would be willing to have more noticeable dental work if it was better for the environment.

☐ Strongly Agree	☐ Agree	☐ Neutral	☐ Disagree	☐ Strongly Disagree

4. I would be willing to have a silver or gold, rather than a white, filling in my front tooth if it was better for the environment.

☐ Strongly Agree	☐ Agree	☐ Neutral	☐ Disagree	☐ Strongly Disagree

5. I would be willing to have a silver or gold, rather than a white, filling in my back tooth if it was better for the environment.

☐	☐	☐	☐	☐
Strongly Agree	Agree	Neutral	Disagree	Strongly Disagree

Health (2 items):

1. I would be willing to compromise the health of my teeth for the environment.

☐	☐	☐	☐	☐
Strongly Agree	Agree	Neutral	Disagree	Strongly Disagree

2. I would not be willing to compromise the health of my teeth for the environment.

☐	☐	☐	☐	☐
Strongly Agree	Agree	Neutral	Disagree	Strongly Disagree
